# *LRRTM4* and *PCSK5* Genetic Polymorphisms as Markers for Cognitive Impairment in A Hypotensive Aging Population: A Genome-Wide Association Study in Taiwan

**DOI:** 10.3390/jcm8081124

**Published:** 2019-07-29

**Authors:** Yi-Chun Chen, Yu-Li Liu, Shih-Jen Tsai, Po-Hsiu Kuo, Shih-Sin Huang, Yun-Shien Lee

**Affiliations:** 1 Department of Neurology, Chang Gung Memorial Hospital Linkou Medical Center and College of Medicine, Chang-Gung University, No.5, Fuxing St., Guishan Township, Taoyuan County 333, Taiwan; 2 Dementia Center, Chang Gung Memorial Hospital Linkou Medical Center, Taoyuan County 333, Taiwan; 3 Center for Neuropsychiatric Research, National Health Research Institutes, 35 Keyan Road, Zhunan, Miaoli County 35053, Taiwan; 4 Department of Psychiatry, Taipei Veterans General Hospital, No. 201, Shih-Pai Road, Sec. 2, Taipei 11217, Taiwan; 5 Division of Psychiatry, National Yang-Ming University, No.155, Sec.2, Linong Street, Taipei, 112 Taiwan; 6 Department of Public Health, Institute of Epidemiology and Preventive Medicine, National Taiwan University, No.17, Xuzhou Rd, Taipei 100, Taiwan; 7 Institute of Statistical Science, Academia Sinica, 128 Academia Road, Section 2, Nankang, Taipei 11529, Taiwan; 8 Department of Biotechnology, Ming Chuan University, 5 De Ming Rd., Taoyuan City 333, Taiwan; 9 Genomic Medicine Research Core Laboratory, Chang Gung Memorial Hospital, No.5, Fuxing St., Guishan Township, Taoyuan County 333, Taiwan

**Keywords:** hypotension, single nucleotide polymorphism, cognitive impairment, dementia

## Abstract

Hypotension can affect cerebral perfusion and worsen cognitive outcomes. The prevalence of low blood pressure (BP) rises with increasing age. To our knowledge, no study has examined the genetic biomarkers for hypotension-related cognitive impairment (CI) yet. Utilizing the population-based genome-wide study of the Taiwan Biobank containing the data of 2533 healthy aging subjects, we found after adjustments for age, sex, education years, and principal components at a suggestive level of 1 × 10^−5^ that minor alleles of *leucine rich repeat transmembrane neuronal 4 (LRRTM4)* (rs13388459, rs1075716, rs62171995, rs17406146, rs2077823, and rs62170897), *proprotein convertase subtilisin/kexin type 5* (*PCSK5)* (rs10521467), and the intergenic variation rs117129097 (the nearby gene: *TMEM132C*) are risk factors for CI in hypotensive subjects. Except for rs117129097, these single nucleotide polymorphisms (SNPs) were not markers per se for CI or for BP regulation. However, we found a suggestive interaction effect between each of the eight SNPs and hypotension on CI risk. In the hypotensive participants, those carrying minor alleles were associated with a higher incidence of CI in an additive manner than were those carrying major alleles (2 × 10^−4^ to 9 × 10^−7^). Intensive BP lowering in elderly patients carrying a minor allele of the eight identified SNPs should raise cautions to prevent a potential treatment-induced neurodegeneration.

## 1. Introduction

Hypotension has been linked to Alzheimer’s disease (AD) [1,2,3], vascular dementia [4], and all-cause dementia [5,6,7]. Blood pressure (BP) starts to decrease approximately three years before a dementia diagnosis [8,9,10]. Longitudinal community-based cohort studies have shown that decline in BP may increase the risk for subsequent development of AD and dementia [8,9,11,12]. Dementia risk was higher in dementia-free elderly (over age 75) who had low BP [8,9,11], especially for those with persistently low BP [11]. A prospective study with a 30-year interval demonstrated that a decrease in systolic BP was related to lower psychomotor speed test in late life [13]. Therefore, low BP and BP reduction may increase the risk of cognitive impairment. Along with rising dementia severity, BP declines gradually. Symptomatic hypotension is present in approximately 30% of all subjects 70 years of age or older [14]. Hypotension is not only a risk factor for cognitive impairment (CI) and cognitive decline but may also be an initial presentation of CI [1,2,3]. Previous studies have shown that hypotension leads to inadequate cerebral perfusion, loss of autoregulation, and endothelial dysfunction in the neurovascular unit [5], which further provokes microvascular disease, stroke, and deposition of amyloid β (Aβ) proteins and neurofibrillary tangles [5,15,16]. A higher incidence of orthostatic hypotension in AD patients than in age-matched non-demented control subjects was noted [17]. Therefore, BP dysregulation may accelerate cognitive decline by lowering the cerebral blood flow. 

According to the definition of the National Heart, Lung, and Blood Institute, hypotension can be defined as a systolic BP (SBP) < 90 mmHg or a diastolic BP (DBP) < 60 mmHg, which are both below the expected values in a healthy individual. Although hypertension has been acknowledged as a risk factor for cardiovascular events and dementia [18,19], the existence of a J-curve has been recognized describing an inverse correlation between DBP levels < 60 mmHg and cardiovascular risks [20]. A similar J-curve phenomenon has also been reported for the correlation of low scores in neuropsychological tests with SBP values below 140 or above 180 mmHg [7,21]. The prevalence of AD was higher in subjects with an SBP/DBP ratio below 130/70 mmHg than in their normotensive counterparts [1]. Both low and high DBP (<60 and >110 mmHg) related to a faster AD progression over a five year follow-up period. However, patients with orthostatic hypotension, cognitive impairment, and multiple comorbidities are at risk of adverse outcomes with intensive BP lowering [22], and the optimal BP for elderly patients with neurodegeneration is controversially discussed [5].

Blood pressure is a highly heritable trait [23]. To date, the susceptible genes for a link between hypotension and CI remain less explored [19]. To identify genetic biomarkers for hypotension-related CI, we analyzed the whole genome data of 2533 aging healthy participants whose cognitive functions were examined as a part of the Taiwan Biobank dataset. The Taiwan Biobank is a prospective population-based study which enrolled 12,000 healthy seniors aged 60–70 years with extensive baseline phenotypic measurements and stored biological samples [24,25]. Here, we report potentially susceptible genetic loci for CI in hypotensive subjects.

## 2. Materials and Methods

### 2.1. Study Population and Phenotypic Data

This study incorporated Taiwanese subjects from the Taiwan Biobank, which gathered the information and specimens from participants in recruitment centers across Taiwan. Inclusion criteria were individuals who were 60 years of age or older and who self-reported as being of Taiwanese Han Chinese ancestry [24]. Participants with a history of cancer were excluded. Ethical approval for the study was granted by the Internal Review Board of the Taiwan Biobank before conducting the study. All experiments were performed in accordance with the relevant guidelines and regulations.

The mean SBP and DBP values were measured based on the average of two seated BP measurements at rest. Pulse pressure was calculated as SBP minus DBP. Hypotension was defined as an SBP < 90 mmHg or a DBP < 60 mmHg. The time course between measurement of BP and cognitive testing was within a day. All participants were healthy elders with no apparent cause of secondary hypotension at the time of recruitment. Because mild CI may involve different cognitive domains of neuropsychological assessments in a wide spectrum, given our prior results in a longitudinal study [26], we utilized a score < 26 (as an average score of amnestic mild cognitive impairment (MCI) and dysexecutive MCI) in the Mini-Mental State Examination (MMSE) as a classification cutoff for CI in this study.

### 2.2. Genome-Wide Association Study (GWAS) and Imputation

Single nucleotide polymorphism (SNP) genotypes were obtained from the data that were derived from the custom Taiwan Biobank chips and run on the Axiom Genome-Wide Array Plate System (Affymetrix, Santa Clara, CA, USA). Quality control procedures for markers including Hardy-Weinberg tests (*p*-value > 0.01), genotype missing rate (≤5%), minor allele frequency (MAF ≥ 0.01), and quality in clustering (good calling) were performed with the genome-wide association study (GWAS) SNP data.

We carried out the discovery GWAS analyses of comparisons between hypotensive participants with CI and normotensive participants with normal cognitive functions. Based on 590,244 SNPs, GWAS was performed using a logistic regression with additive models by a whole genome data analysis toolset, PLINK version 1.9 [27,28], adjusting for age, sex, education years, and principal components (PCs). Adjustments for population stratification with top 10 principal components were also performed using PLINK. SNPnexus (https://www.snp-nexus.org/index.html) and the R package VariantAnnotation were used to annotate the function of SNPs to NCBI RefSeq genes.

For clustered SNPs with P < 1 × 10^−5^, we used imputation by searching the functional SNPs using the PLINK imputation command with the population-scale sequencing database, 1000 Genomes Phase III NCBI Build b37 (May 2014) reference panel (http://ftp.1000genomes.ebi.ac.uk/vol1/ftp/phase3/). The approach works by finding haplotype segments that are shared between individuals in the Taiwan Biobank and a reference panel (1000 Genomes) including a combined of 312 CHB (Han Chinese in Beijing, China), CHS (Southern Han Chinese), and JPT (Japanese in Tokyo, Japan) individuals. Imputation methods can accurately estimate genotypes at markers that have not been directly examined in a GWAS to guide fine-mapping efforts. 

We first estimated the haplotypes for each individual within the GWAS sample (pre-phasing) and then imputed missing genotypes into these estimated haplotypes. SNPs were subsequently removed if the imputation quality score was less than 0.8 or the MAF was less than 0.01. We calculated the D′ values with the Haploview software (version 4.1) to estimate the haplotype of the SNPs in the samples.

### 2.3. Statistics

Linkage disequilibrium was computed for each tandem pair of SNPs and was estimated as D’. The χ^2^ test or t-test was utilized to compare demographic data. The P-value of statistical significance was adjusted by Fisher’s exact test where appropriate; all significance tests were two-tailed. For genotype-phenotype association analyses, we assumed an additive model of inheritance. We conducted multiple logistic regression analyses to test the null hypothesis that the number of cases and controls did not differ by increasing minor allele copy number. For continuous variates, general linear models (GLM) were applied. The associations of hypotension, genotypes, and hypotension-genotype interaction with CI were examined. Potential covariables included age, sex, education years, and PCs. 

## 3. Results 

In the 2533 healthy aging subjects of the Taiwan Biobank dataset, there was no difference in age, education years, proportion of diabetes mellitus, habit of alcohol drinking, and heart rate between the hypotensive (*n* = 166) and non-hypotensive (*n* = 2367) groups (Table 1). Study participants of female sex and with lower Body mass index (BMI) were particularly prone to have hypotension (*p* < 0.0001). The proportion of self-reported hypertension history was lower in the hypotensive (12.1%) than in the non-hypotensive group (27.7%). In the entire cohort, 24.6% of the participants presented a cognitive impairment (MMSE < 26). Compared to the non-hypotensive group, the hypotensive group exhibited a higher proportion of CI (24.0% vs. 33.7%, non-hypotensive vs. hypotensive group, respectively; *p* = 0.0049), and this result remained statistically suggestive after adjusting for age, sex, education years, and smoking (*p* = 0.029). 

### 3.1. Discovery SNP-Based Association Analysis to Explore Candidate SNPs for Hypotension-Related CI

**Figure 1:**
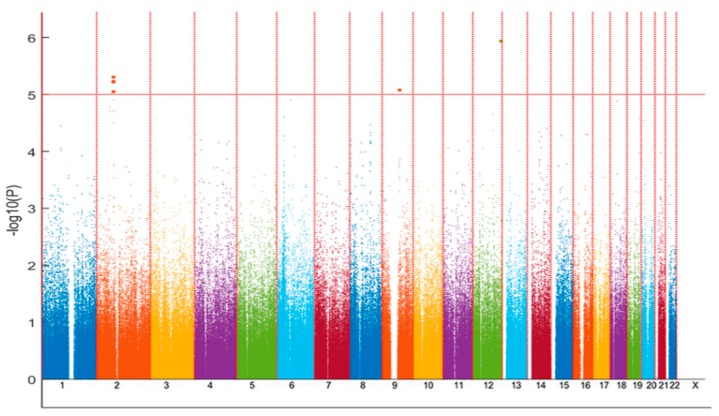
Manhattan plots of the genome-wide association scan. Manhattan plots of the genome-wide association scan for regions associated with the coexistence of cognitive impairment (CI) and hypotension shows the clusters of suggestive single nucleotide polymorphisms (SNPs) within the loci of chromosome 2p12, 77.09 M to 77.33 M, and two spots at chromosome 9q21.13 and 12q24.32 at a significance level of 1 × 10^−5^. Eight SNPs within three genes (six in *leucine rich repeat transmembrane neuronal 4 (LRRTM4)*, one in *proprotein convertase subtilisin/kexin type 5* (*PCSK5)*, and one unknown (the nearby gene is *TMEM132C*)) were identified to be associated with the concurrent presence of hypotension and CI.

**Figure 2:**
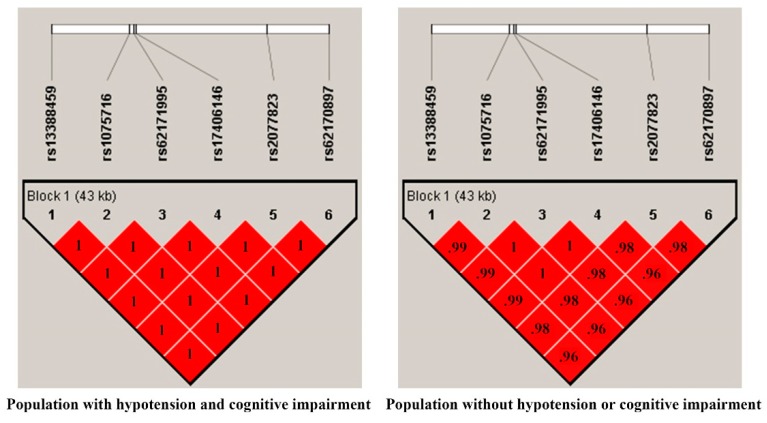
Linkage disequilibrium coefficients (D’) of the pairwise loci constructed by the six SNPs in *LRRTM4*. Linkage disequilibrium coefficients (D’) of the pairwise loci constructed by the six SNPs in *LRRTM4* show no difference between cases and controls (Haploview version 4.2 software). A D’ value of “1” indicates that the examined two loci exhibit a complete linkage while a value of “0” demonstrates their independence. The most common haplotypes were CTGAAA with 69.9% and TCAGGGG with 30.4% in the group with coexisting hypotension and cognitive impairment, whereas 85.7% CTGAAA and 13.4% TCAGGGG were determined in the control group.

For the discovery SNP-based association analysis, we compared first subjects with coexisting hypotension and CI (*n* = 56) and those without hypotension and normal cognition (normal control, NC, *n* = 1799) after adjusting for age, sex, education years, and PCs. Eight SNPs within three genes (rs13388459, rs1075716, rs62171995, rs17406146, rs2077823, and rs62170897 in *leucine rich repeat transmembrane neuronal 4 (LRRTM4)*; rs10521467 in *proprotein convertase subtilisin/kexin type 5* (*PCSK5)*; and rs117129097 in an intergenic region) were associated at a suggestive level of 1 × 10^−5^ with coexisting hypotension and CI (Figure 1 and Table 2). Because smoking habits influenced hypotension status, we tested if smoking habits influenced the associations between SNPs and CI status by adding smoking as an adjusted variable in logistic regression. However, because sex and smoking were highly correlated with each other (χ^2^ test, *p* < 1E-196, odds ratio = 27.2), that is, both variables are multicollinearity, only one of the two variables should be included in the regression model at one time [29]. Therefore, we further examined the association analysis after adjusting for age, education years, PCs, and smoking, and similar results were yielded as the model adjusted for sex (Table 2). Each of the SNPs was in Hardy-Weinberg equilibrium (significance level, 0.01). The cluster of the six *LRRTM4* SNPs within the loci of chromosome 2 77.09 M to 77.33 M locates in the same block (D’: 0.97–1 among the SNPs) (Figure 2). Within the associated loci, we took forward for genotype imputation according to the information of linkage disequilibrium to search for the functional SNPs associated with the presence of coexisting hypotension and CI. Imputation was performed using the PLINK imputation command with the population-scale sequencing database, the 1000 Genomes Phase III NCBI Build b37 reference panel. This approach finds haplotype segments that are shared between individuals in the Taiwan Biobank and in the 1000 genomes including 197 samples. Imputation analyses, however, did not reveal further nonsynonymous or functional SNPs associated with the coexistence of hypotension and CI. 

### 3.2. Associations of The Eight Identified SNPs with Hypotension or with CI in The Whole Cohort

Further evaluations for how the discovered SNPs were associated with CI or with hypotension in the entire study population showed weak associations between the six established *LRRTM4* SNPs and CI when adjusted for age, sex, education years, and PCs (Table 3). There was no association of CI with *PCSK5* rs10521467 and rs117129097 at the intergenic region of the unknown gene. In contrast, rs117129097 was associated with hypotension when adjusted for age, sex, education years, and PCs (odds ratio = 1.90, 95% CI: 1.33–2.72, *p* = 0.0005). There was no association between hypotension and any of the remaining seven SNPs. Because the adjustment for sex or smoking as confounding factors yielded similar results, Table 3 showed only the results of adjustment of age, sex, education, and PCs.

### 3.3. Interaction Effects of SNPs and Hypotension on CI in the Whole Cohort 

**Figure 3:**
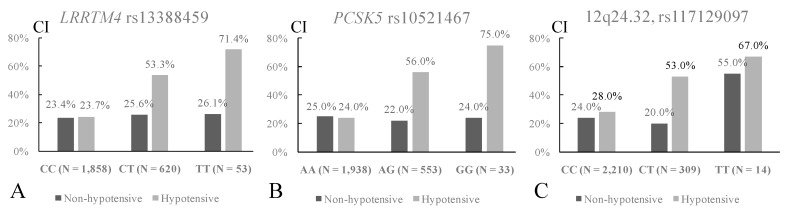
Interactive effects of the discovered SNPs and hypotension on CI in the whole cohort. There was a suggestive interactive effect between rs13388459 and hypotension on cognitive impairment (CI) risk (*p* = 0.0004). Hypotensive subjects carrying the rs13388459 T allele were associated with a higher incidence of CI in an additive manner compared to those carrying the C allele (panel A). By contrast, in the non-hypotensive group, there was no difference in CI incidence between the rs13388459 genotypes. Similarly, there was a suggestive interaction effect between *PCSK5* rs10521467 and hypotension on CI risk (*p* = 9 × 10^−7^; panel B). The SNP rs117129097 was associated with hypotension (*p* = 0.0005). An additional suggestive interaction effect was found between rs10521467 and hypotension on CI risk (*p* = 0.003; panel C).

While the *LRRTM4* SNPs were not associated with hypotension or CI, we found a suggestive interaction effect between each of these six SNPs and hypotension on CI risk. The analysis revealed that hypotensive subjects carrying the rs13388459 T allele were associated in an additive manner with a higher CI incidence compared to those carrying the C allele (Figure 3A). By contrast, in the non-hypotensive population, there was no difference in CI incidence between the rs13388459 genotypes (Figure 3A). The interaction effects of the other *LRRTM4* SNPs and hypotension on CI were in the same significance range as that of rs13388459, in which the significance levels of interactive effects for rs1075716, rs62171995, rs17406146, rs2077823, and rs62170897 were 8 × 10^−6^, 9 × 10^−6^, 6 × 10^−5^, 2 × 10^−4^, and 2 × 10^−4^, respectively. Additionally, there was suggestive interaction effects of the *PCSK5* rs10521467 and intergenic SNP rs117129097 and hypotension on CI risk (*p* = 9 × 10^−7^; Figure 3B and *p* = 3 × 10^−3^; Figure 3C, respectively). 

## 4. Discussion

This population-based GWAS demonstrated a suggestive correlation between hypotension and cognitive impairment in healthy aging participants. The current study identified *LRRTM4* (rs13388459, rs1075716, rs62171995, rs17406146, rs2077823, and rs62170897), *PCSK5* (rs10521467), and the intergenic SNP rs117129097 as markers for CI coexisting with hypotension. Except for rs117129097 that was associated with hypotension, these identified SNPs may not be suggestive markers per se for CI or for BP regulation. Although there were weak associations between the six established *LRRTM4* SNPs and CI, the significance may not survive when considering multiple-testing issues. The elderly people carrying minor alleles of the eight identified SNPs are susceptible to CI when they also have hypotension. Therefore, excessive decrease in the BP in those carrying the minor alleles of the eight described SNPs should particularly raise cautions to prevent a potential treatment-induced neurodegeneration [5].

Inadequate cerebral blood flow caused by microvascular deficits leads to diminished brain supply of oxygen, energy, substrates, and nutrients, especially due to age-related alterations in the cerebral autoregulation [16]. Neurovascular uncoupling may induce neurodegeneration [16,30]. The mechanisms of hypotension-related CI are considered to relate to inadequate cerebral perfusion, loss of autoregulation, and endothelial dysfunction in the neurovascular unit, which leads to microvascular disease, stroke, and deposition of Aβ protein and neurofibrillary tangles [5]. Clearance of Aβ from the brain is dependent on vascular reactivity, which in turn is affected by microvascular disease [16,30].

Leucine-rich-repeat transmembrane neuronal proteins (LRRTMs) are a family of four synapse-organizing proteins involved in protein–protein interactions and critical for regulating the development and function of excitatory synapses. The genes encoding LRRTMs are associated with multiple psychiatric disorders [31]. A quantitative transcriptomics analysis (RNA-Seq) showed that *LRRTM4* is particularly expressed in the central nervous system [32]. *LRRTM4* mRNA is highly expressed in most brain regions, including the olfactory bulb, striatum, inferior colliculi, and dentate gyrus [33]. The structure and expression profile of *LRRTM* mRNAs suggest that these proteins may have a role in the development and maintenance of the nervous system [33]. LRRTM4 has distinct presynaptic binding partners, the heparan sulfate proteoglycans (HSPGs). HSPGs are known to mediate the synaptogenic activity of LRRTM4 [34]. Within the hippocampus, LRRTM4 was detected specifically at excitatory postsynaptic sites of dentate gyrus granule cells [34]. Dentate gyrus granule cells of *Lrrtm4* knockout animals exhibit a reduced excitatory synapse density and function and an impaired activity-regulated AMPA receptor trafficking. Our study reveals the interaction effects of *LRRTM4* SNPs and hypotension on CI suggesting that a failed maintenance of synapse organization may occur during cerebral hypoperfusion. 

The *PCSK5* gene encodes the proprotein convertase subtilisin/kexin type 5 (PCSK5) which belongs to the PCSK family that processes peptide precursors and regulates the functions of numerous molecules [35]. Members of the PCSK family modulate the activity of precursor proteins and are particularly related to lipid and insulin metabolism [35] as well as the BP trait [36,37]. Alternative splicing in *PCSKs* results in multiple transcript variants. Mutations in *PCSK5* may lead to abnormal metabolism of high-density lipoprotein through dysregulation of signal molecules in the bile acid. PCSK5 influences the lipoprotein metabolism by modulating the activity of endothelial lipases, lipoprotein lipases, and the level of low-density lipoprotein receptors through PCSK9 cleavage [35]. To date, there are several reports presenting evidence for an association of *PCSK5* with BP trait [36,37]. The report herein supported the association of *PCSK5* and CI risks in hypotensive subjects. Whether the genetic variation related to lipid metabolism causes an arterial dysfunction needs to be verified in future studies. By contrast, the intergenic SNP rs117129097 is associated with hypotension and plays a suggestive interaction effect with hypotension on CI risk. To date, the function of this SNP is unknown.

The present study utilized a GWAS to identify eight SNPs as susceptible genetic variants for CI in the presence of hypotension. However, there are several limitations of this study. First, the number of cases is relatively small. Although the effect sizes are moderate in the studied population, the presented results need to be replicated before these SNPs can be viewed as independent risk factors for hypotension-related cognitive impairment. Second, this study did not include information about BP variation, continuous BP monitoring, or details regarding the used medications, which may influence the hypotension categorization of the study participants. Third, the Taiwan Biobank did not list dementia as an exclusion criterion, which may limit the generalizability of our findings. In addition, because the Taiwan Biobank did not primarily aim to identify dementia population, there was no adequate information to clarify the causes of CI in our study. Forth, because we did not have medication lists, the categorization of the sample on the basis of the drugs was not able to be performed. Fifth, the identified SNPs are intron variations, which may not be the causal SNPs to explain the pathophysiology of CI. Measuring the expression levels of these genes may provide additional functional information to support our hypothesis. Further replication studies addressing these limitations in other ethnic populations are needed to confirm the results presented in this study.

## Figures and Tables

**Table 1:** Demographic data of 2,533 clinically normal elderly subjects.

Variable	Non-Hypotensive *n* = 2367	Hypotensive *n* = 166	*p*-Value
Age (years)	64.01 ± 2.89	64.43 ± 2.95	0.07
Male sex (*n*, %)	1243, 52.5	45, 27.1	<0.0001
Education (years)	4.91 ± 1.24	4.80 ± 1.40	0.24
Self-reported HTN (*n*, %)	656, 27.7	20, 12.1	<0.0001
Self-report DM (*n*, %)	267, 11.3	23, 13.9	0.31
Alcohol (*n*, %)	158, 6.7	5, 3.0	0.06
Smoking (*n*, %)	736, 31.1	35, 21.1	0.007
Body mass index (kg/m^2^)	24.54 ± 3.10	22.82 ± 3.05	<0.0001
Resting SBP (mmHg)	133.57 ± 19.84	108.74 ± 15.97	<0.0001
Resting DBP (mmHg)	78.39 ± 11.28	56.42 ± 5.04	<0.0001
Pulse pressure (mmHg)	55.18 ± 13.78	52.33 ± 14.6	0.015
HR (/min)	69.46 ± 9.16	68.58 ± 9.40	0.235
MMSE < 26 (*n*, %)	568, 24.0	56, 33.7	0.0049, 0.029 *

HTN: hypertension; DM: diabetes mellitus; SBP: systolic blood pressure; DBP: diastolic blood pressure; HR: heart rate; MMSE: Mini-Mental State Examination. Comparisons between groups were analyzed using the χ^2^ test, Fisher's exact test, or t-test, where appropriate. * *p*-value derived by logistic regression, adjusted for age, sex, education years, and smoking.

**Table 2:** Results for SNPs related to hypotensive cognitive impairment (CI; *n* = 56) compared to non-hypotensive, normal cognitive controls (NC, *n* = 1,799).

Gene	Locus	SNP	Position	A1/A2	MAF (hypotensive CI/NC)	OR (95% CI), *p*-Value, *p*-Value *
*LRRTM4* (intron variations)	2p12	rs13388459	77215497	T/C	0.30/0.14	2.85 (1.81–4.49), 6.07 × 10^−6^, 6.08 × 10^−6^
		rs1075716	77227586	C/T	0.30/0.14	2.85 (1.81–4.49), 5.99 × 10^−6^, 5.96 × 10^−6^
		rs62171995	77228320	A/G	0.30/0.14	2.86 (1.81–4.50), 5.86 × 10^−6^, 5.79 × 10^−6^
		rs17406146	77228667	G/A	0.30/0.14	2.84 (1.81–4.46), 5.97 × 10^−6^, 6.48 × 10^−6^
		rs2077823	77248912	G/A	0.30/0.14	2.88 (1.83–4.53), 4.99 × 10^−6^, 4.49 × 10^−6^
		rs62170897	77258540	G/A	0.30/0.14	2.78 (1.77–4.36), 9.01 × 10^−6^, 6.78 × 10^−6^
*PCSK5* (intron variation)	9q21.13	rs10521467	78651491	G/A	0.27/0.12	2.94 (1.83–4.75), 8.41 × 10^−6^, 1.94 × 10^−5^
Unknown (intergenic region)	12q24.32	rs117129097	128539282	T/C	0.19/0.06	4.03 (2.30–7.08), 1.17 × 10^−6^ 1.56 × 10^−6^

SNP: single nucleotide polymorphism; A1: minor allele; A2: major allele; MAF: minor allele frequency; OR: odds ratio; 95% CI: 95% confidence interval. *p*-values derived by logistic regression, adjusted for age, sex, education years, and principal components (PCs). * *p*-values derived by logistic regression, adjusted for age, smoking, education years, and PCs.

**Table 3:** Associations of identified SNPs with hypotension and with cognitive impairment (CI) in the whole cohort.

			Hypotensive (*n* = 166)/Non-Hypotensive (*n* = 2,367)		CI (*n* = 624)/Non-CI (*n* = 1,909)	
Gene	SNP	A1/A2	MAF	OR (95% CI), *p*-Value	MAF	OR (95% CI), *p*-Value
*LRRTM4*	rs13388459	T/C	0.18/0.14	1.32 (0.98–1.77), 0.07	0.16/0.14	1.22 (1.01–1.48), 0.04
	rs1075716	C/T	0.18/0.14	1.32 (0.98–1.77), 0.07	0.16/0.14	1.22 (1.00–1.48), 0.05
	rs62171995	A/G	0.18/0.14	1.32 (0.98–1.77), 0.07	0.16/0.14	1.23 (1.02–1.49), 0.03
	rs17406146	G/A	0.18/0.14	1.30 (0.97–1.75), 0.08	0.16/0.14	1.23 (1.01–1.49), 0.04
	rs2077823	G/A	0.18/0.14	1.34 (1.00–1.80), 0.05	0.17/0.14	1.27 (1.05–1.54), 0.02
	rs62170897	G/A	0.18/0.14	1.29 (0.96–1.73), 0.09	0.17/0.14	1.27 (1.05–1.54), 0.01
*PCSK5*	rs10521467	G/A	0.15/0.12	1.35 (0.98–1.86), 0.06	0.13/0.12	1.05 (0.85–1.30), 0.66
Unknown *	rs117129097	T/C	0.11/0.06	1.90 (1.33–2.72), 0.0005	0.07/0.07	1.08 (0.82–1.43), 0.57

SNP: single nucleotide polymorphism; A1: minor allele; A2: major allele; MAF: minor allele frequency; OR: odds ratio; 95% CI: 95% confidence interval. * rs117129097 at chr12:128539032–128539532, the nearby gene is *TMEM132C* chr12:128,751,948–129,192,460. *p*-values derived by logistic regression, adjusted for age, sex, education years, and PCs.
